# Treatment of a Gustilo-Anderson type II open tibia shaft fracture with an ultrathin silver plasma-coated plate: A case report

**DOI:** 10.1016/j.tcr.2022.100715

**Published:** 2022-10-17

**Authors:** Volker Alt, Christian Pfeifer, Markus Rupp, Nike Walter

**Affiliations:** Department of Trauma Surgery, University Hospital Regensburg, Regensburg, Germany

**Keywords:** Silver, Coating, Fracture-related infection, Antimicrobial implant, Open fracture

## Abstract

The treatment of open tibia fractures with plate fixation bears a high risk for the development of a fracture-related infection. Here we present a case of an 80-year-old female with a Gustilo-Anderson type II open distal tibial shaft fracture with an indwelling knee prosthesis. The case was successfully treated with a 2-stage procedure with initial external fixation and subsequent open reduction and plate fixation with a custom-made ultrathin silver plasma-coated locking plate. Plate fixation was the preferred method as the indwelling knee prosthesis prevented the use of a standard intramedullary tibia nail. The fracture healed uneventfully without any signs of infections and the patient achieved full weight-bearing with a normal gait and without any pain after three months. Radiographical full bridging of the initial fracture area was seen after 5 months without any signs of infection or fracture healing disturbances. After an overall follow-up of 12 months, there were no signs of silver associated or other adverse events. Thus, ultrathin silver plasma coating seems to be helpful in the treatment open fractures with plate fixation to prevent fracture-related infections.

## Introduction

The treatment of open fractures can be challenging bearing the risk of the development of a fracture-related infection and associated impaired bone consolidation. In recent years, the role of antimicrobial coating of implants to circumvent side effects of systemic antibiotic therapy while at the same time achieving higher local concentration of the antimicrobial agent has become increasingly important. Hitherto, multiple individually antibiotic cement coatings and distinct implants such as gentamicin coated intramedullary nails are commercially available [1], [2]. Silver has been introduced as a promising antimicrobial coating option being independent of antibiotic resistance, which is an increasing concern worldwide [3]. Different types of silver-coated endoprotheses have been used for the treatment and prevention of periprosthetic joint infection as well as for oncological limb salvage surgery [4], [5], [6]. Recently, also a first report of a case with infected long bone non-union successfully managed with a gamma nail coated with ultrathin silver plasma was published [7]. Here, we report, for the first time, the use of a custom-made ultrathin silver-coated locking plate for fixation of a Gustilo-Anderson type II open tibia shaft fracture to prevent fracture-related infection.

## Clinical case

**Fig. 1:**
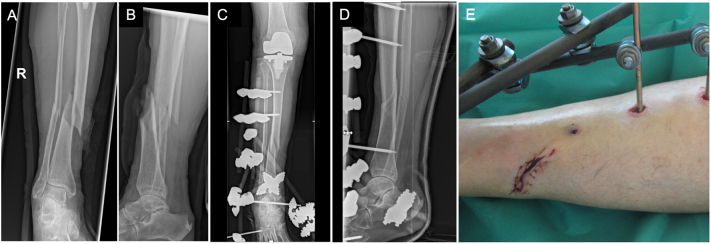
Anterior-posterior (A) and lateral (B) X-ray of the distal spiral tibia shaft fracture (AO type 42-A1) with concomitant fibula shaft fracture of the right leg. Postoperative anterior-posterior X-ray after closed reduction and external fixation (C). Photograph of the primarily sutured wound with a length of 5 cm directly over the fracture site (D).

**Fig. 2:**
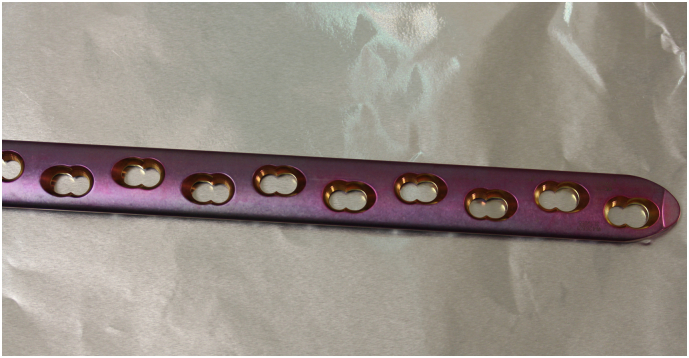
Ultrathin silver plasma-coated (HyProtect®, Bio-Gate AG, Nürnberg, Germany) 12-holes LCP (DePuy Synthes®, Oberdorf, Switzerland) before implantation, which shows a slight modification of colour of the plate from gold to purple due to the plasma polymer coating process. (For interpretation of the references to colour in this figure legend, the reader is referred to the web version of this article.)

**Fig. 3:**
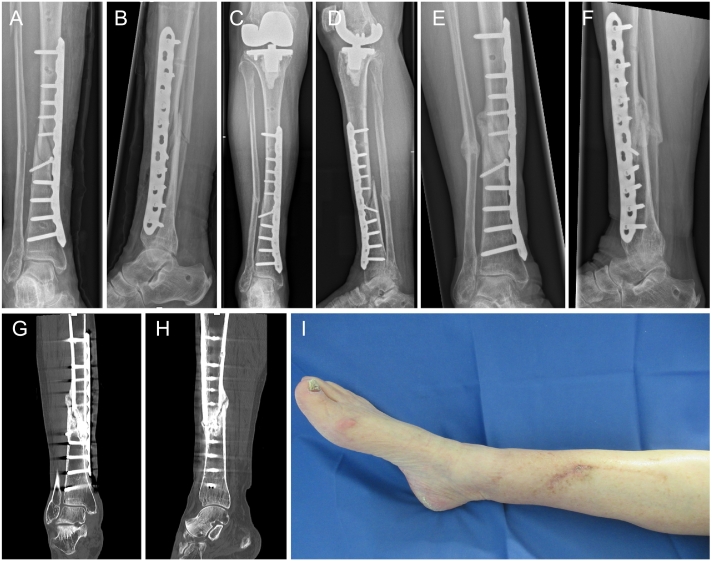
Anterior-posterior (Ap) (A) and lateral (B) X-ray after open reduction, plate and lag screw fixation of the distal tibia shaft fracture. Ap (C) and lateral (D) X-rays at 6 weeks with first signs of callus formation. Ap (E) and lateral (F) X-rays at 5 months with bridging callus formation and full consolidation of the tibia and of the fibula fracture confirmed by representative coronal (G) and sagittal (H) CT sections. Clinical image of the distal leg (I) showing undisturbed wound and soft tissue healing at 5 months without any signs of argyria (blue-grey decolorization of the skin around the silver-coated plate) or other silver-specific side effects. (For interpretation of the references to colour in this figure legend, the reader is referred to the web version of this article.)

An 80-year-old female (BMI 26 kg/m^2^) sustained a fall from the stairs and sustained a Gustilo-Anderson type II open tibial shaft spiral fracture (AO type 42-A1) with a concomitant fracture of the fibula shaft of the right leg (Fig. 1). Emergency treatment consisted of débridement of the open fracture site, reduction of the fracture, primary wound closure and application of an external monotube fixator. Three times 2 g i.v. cefazolin was administered for three days. Pre-operative planning for the stage 2 intervention revealed a 4.5 mm 12-holes LCP (DePuy Synthes®, Oberdorf, Switzerland) to be appropriate for plate fixation of the fracture and this plate was shipped for ultrathin silver plasma coating (HyProtect®, Bio-Gate AG, Nürnberg, Germany) to the external manufacturer (Bio-Gate AG, Nürnberg, Germany) (Fig. 2). The coated plate was received back after 10 days. There was undisturbed wound healing and on post-operative day 16, the fixator was removed and the custom-made ultrathin silver plasma coated locking plate was applied. Informed consent for the use of the silver-coated implant was pre-operatively given by the patient. Upon removal of the fixator, the wound was re-opened and débrided again. After open reduction of the fracture, a 4.5 mm lag screw was inserted for interfragmentary compression. The silver-coated plate was applied in a minimally-invasive technique with a small 5 cm incision over the medial malleolus and a second incision over the midshaft of the tibia that included the initial wound with an extension of another 6 cm to the tibia midshaft. Three 5.0 mm locking screws and two 4.5 cortical screws were placed in the proximal and four 5.0 mm locking screws were placed in the distal part of the 12-holes LCP. Postoperative X-rays showed a good reduction of the fracture and correct positioning and length of the plate and screws (Fig. 3). The patient was allowed partial weight-bearing of 20 kg for six weeks followed by full weight bearing. The wounds healed without any further complications and the patient could be discharged after 28 days post initial trauma.

The patient showed undisturbed wound healing and first signs of callus formation fracture at six weeks (Fig. 3). Full weight-bearing with normal gait and without pain was seen at 12 weeks. Radiographic fracture consolidation of the fracture with full bridging of all 4 cortical areas on ap and lateral X-rays as well as on CT scans was achieved within five months without any complications, such as signs of infection or delayed fracture healing (Fig. 3). Thus, no further surgical intervention or antibiotic treatment was required. In addition, no silver associated adverse events, such as argyria presenting with blue-grey decolorization of the skin, occurred 12 months after the intervention.

## Discussion

To the best of our knowledge, this is the first case reporting the use of an ultrathin silver plasma coating of a plate for the prevention of a fracture-related infection (FRI). The underlying injury was a Gustilo-Anderson type II open tibia-fibula fracture of the distal shaft area with an indwelling ipsilateral knee prosthesis. The case was successfully managed by a two stage procedure with initial external fixation. Intramedullary nailing and not plate fixation would have been the preferred option for internal fixation at stage 2 in a Gustilo-Anderson type II open tibia fracture due to lower overall complication rates compared to plating, including non-union and infection [8]. However, standard intramedullary tibial nailing was not possible in the current case due to the indwelling knee prosthesis and plate fixation was deemed appropriate. In order to decrease risks for infection and subsequent fracture healing disturbances, antimicrobial coating with a custom-made ultrathin silver plasma coating of the locking plate was advocated.

The ultrathin silver plasma coating used in the present case has several advantages. These include the broad antimicrobial effects of silver against gram-positive pathogens such as *Staphylococcus aureus* and *Staphylococcus epidermidis*, which are the most common microorganisms causing fracture-related infections, as well as against gram-negative strains and fungi [9], [10]. Thus, silver-coated implants are beneficial in cases with polymicrobial contamination in open fractures or FRIs. Further, the coating can be applied to different surfaces including titanium, stainless steel and polymers [11]. In addition, no risk exists regarding the emergence of resistant strains as it might occur when using local gentamicin application [3]. Besides, the technology implements an ultrathin siloxane layer directly on the implant surface (10 to 30 nm), from which a low amount of silver ions is released [11]. The very low concentration of silver reduces the probability of silver specific side effects such as argyria or potential fracture healing disturbances. Here, no silver-adverse events occurred and the fracture went to uneventful and timely consolidation. In line, this was also the case in a previous published reports using an ultrathin plasma silver-coated nail to treat an infected non-union [7] as well as silver-coated knee arthrodesis implant [10], [12].

## Conclusions

The following learning point could be demonstrated based on this case:

1. Fracture fixation plates can be coated with an ultrathin silver plasma coating for the prevention of fracture-related infections
2. The released silver ions did not have a negative effect on bone healing and the fracture consolidated uneventfully within 5 months without any signs of fracture healing disturbances or infection
3. No other silver specific side effects, such as argyria, occurred at 12 months

## Declaration of competing interest

Volker Alt is a consultant for Bio-Gate AG, Nürnberg, Germany.
